# Impact of Dietary Interventions on the Human Plasma and Lipoprotein Lipidome

**DOI:** 10.3390/metabo15090602

**Published:** 2025-09-09

**Authors:** Rosa Casas, Nancy D. Sánchez-García, Ramon Estruch, Anallely López-Yerena

**Affiliations:** 1 Department of Internal Medicine, Hospital Clínic, Institut d’Investigació Biomèdica August Pi i Sunyer, University of Barcelona, 08036 Barcelona, Spain; rcasas1@recerca.clinic.cat (R.C.); restruch@ub.edu (R.E.); 2 Centro de Investigación Biomédica en Red (CIBER) de Fisiopatologia de la Obesidad y la Nutrición (CIBEROBN), Instituto de Salud Carlos III, 28029 Madrid, Spain; 3 Institut de Recerca en Nutrició i Seguretat Alimentaria (INSA-UB), University of Barcelona, 08921 Barcelona, Spain; 4 Polyphenol Research Group, Departament of Nutrition, Food Science and Gastronomy, Faculty of Pharmacy and Food Science, University of Barcelona, 08028 Barcelona, Spain; nsanchga101@alumnes.ub.edu

**Keywords:** Lipidome, LDL, HDL, serum, metabolic health, nutritional modulation

## Abstract

Lipids are structurally diverse biomolecules that play essential roles in cellular function, energy storage, and signaling. The human lipidome, a dynamic and complex subset of the metabolome, is shaped by both endogenous factors, such as genetics, sex, age, and metabolic health, and exogenous influences like lifestyle, diet, and microbiota. Among these, diet stands out as one of the most modifiable and impactful determinants, influencing lipid composition across plasma, serum, and lipoprotein fractions. While traditional lipid profiling provides limited insight, lipidomics enables comprehensive characterization of lipid species, revealing mechanistic links between lipid metabolism and diseases such as cardiovascular disease (CVD), metabolic syndrome (MetS), and inflammatory disorders. This review explores: (1) the relationship between lipid profiles and CVD risk, (2) the internal and external modulators of the lipidome, and (3) current evidence on how specific dietary patterns, including Mediterranean, Nordic, low glycemic, and vegetarian diets, and individual nutrients such as omega-3 fatty acids (FAs), plant sterols, and mycoprotein, influence lipidomic profiles. Advances in lipidomics highlight that dietary fat quality, food matrix, and eating patterns can significantly modulate lipid species such as triacylglycerols (TAGs), ceramides (Cers), and phospholipids, with implications for cardiometabolic health. Notably, distinct responses are observed across plasma High-Density Lipoprotein (HDL) and Low-Density Lipoprotein (LDL) lipidomes, emphasizing the need for compartment-specific analyses. Understanding these diet-lipidome interactions offers promising avenues for precision nutrition and the development of lipid-based biomarkers for disease prevention and management.

## 1. Introduction

Lipids are hydrophobic or amphipathic molecules with distinct structural and biological properties, originating from either endogenous or exogenous sources. These are usually categorized into eight main categories (Table 1): fatty acyls, glycerolipids, glycerophospholipids, sphingolipids, sterol lipids, prenol lipids, saccharolipids (glycolipids), and polyketides, each with further classes and subclasses based on their chemical structure [1]. Lipids such as triacylglycerols (TAGs), diacylglycerols (DAGs), phosphatidylcholines (PCs), phosphatidylethanolamines (PEs), ceramides (Cers), sphingomyelins (SMs) and cholesterol esters (CEs) each consist of a specific backbone architecture conjugated to various fatty acids (FAs) [2].

Although part of the metabolome, the lipidome constitutes a distinct subset of interconnected compounds with critical roles in cell structure, energy homeostasis, cellular signaling, and disease mechanisms [2,3]. The human lipidome is highly dynamic and influenced by endogenous factors such as genetic polymorphisms, enzyme activity, hormonal regulation, and aging. It is also shaped by exogenous factors such as diet, gut microbiota, physical activity, and environmental exposures (e.g., toxins, medications) [4,5,6,7,8]. Among these factors, dietary intake is the most potent and modifiable, as it directly provides lipid precursors and modulates metabolic pathways. In particular, FAs and lipid-rich foods significantly alter human lipidomic profiles by changing the composition and metabolism of various lipid species, such as TAGs, sphingolipids, and polyunsaturated fatty acids (PUFAs) [9,10].

Conventional lipid profiling measures traditional lipid classes, including cholesterol transported by low- and high-density lipoprotein, total triglycerides, and total cholesterol. However, this approach fails to capture the diversity and functional complexity of the lipidome, which comprises numerous structurally and biologically distinct lipid species [9]. The changes in lipids due to metabolic reprogramming have been revealed to be closely associated with a wide range of conditions, including metabolic disorders, immune dysfunction and neurodegenerative diseases, as well as different cancers [10]. Therefore, lipidomics has emerged as a powerful approach to better understanding lipid biology, offering valuable information for understanding lipid biology, and offering new avenues for disease diagnosis, risk prediction and therapeutic targeting. In the context of cardiovascular diseases (CVD), specific lipid species including CEs, lysophosphatidylcholines (LPCs), PCs, PEs, Cers, SMs and TAGs potentially improve CVD risk stratification beyond what is achievable with traditional lipid markers [11,12,13,14,15,16].

Building upon this foundation, the present review explores three critical dimensions of the human lipidome: (1) the relationship between lipid profiles and CVD risk, (2) the key endogenous and exogenous factors that shape lipidome dynamics, and (3) the specific impact of diet on the lipid composition in plasma, serum, and lipoprotein fractions. This tripartite analysis provides a comprehensive framework for understanding nutritional regulation of lipid metabolism and its clinical implications for cardiovascular health.

## 2. Review Methodology

For the purposes of this study, the inclusion criteria were limited to studies conducted in human subjects without time restriction and those written in the English language. A comprehensive search was conducted utilizing the Scopus, PubMed, and Google Scholar databases to identify clinical trials investigating the impact of diet on the lipid composition in plasma, serum, lipoproteins fractions and erythrocyte membranes. The search strategy included the use of the following keywords: lipidome, lipidomics, lipid profiling, biomarkers, plasma-lipidome, high-density lipoprotein (HDL), low-density lipoprotein (LDL) lipidomes, diet, dietary patterns, foods, high-fat foods, cardiometabolic health and CVDs.

## 3. Factors Affecting the Human Lipidome

**Figure 1:**
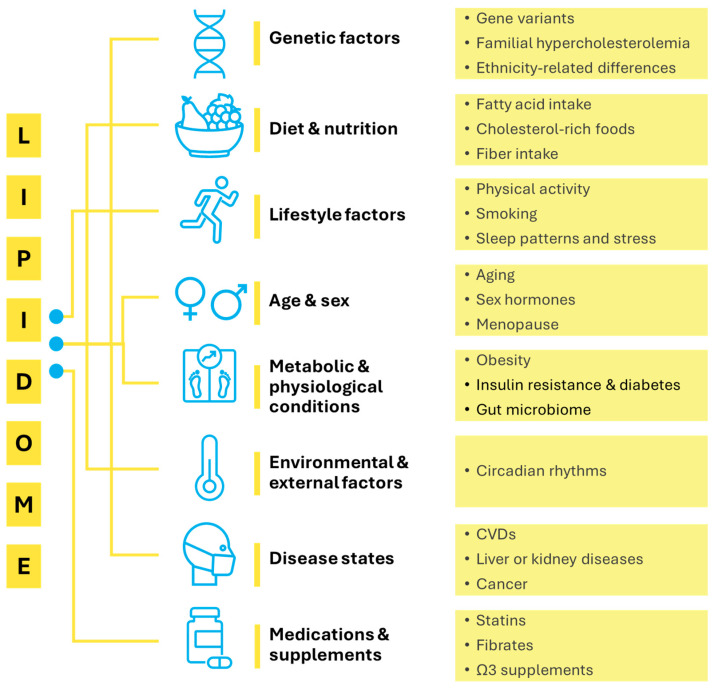
Factors affecting human lipidome.

The human lipidome is highly dynamic and shaped by a variety of intrinsic (endogenous) and extrinsic (exogenous) factors that interact to influence lipid profiles and associated health outcomes (Figure 1). Because lipids are metabolically interconnected, changes in one of the components of the system often cascade though related lipid classes and pathways.

### 3.1. Genetic and Physiological Determinants

Genetic factors significantly influence the human lipidome profile, with several studies demonstrating substantial heritability and identifying specific genomic loci associated with distinct lipid species [4,5,6]. These genome-wide association studies have identified thousands of single nucleotide polymorphisms (SNPs) for specific genes that can explain a significant fraction of the variation in lipid profiles. Studies confirm that the lipidome is heritable, with heritability varying among different lipid classes; for example, Cers have higher heritability compared to phosphatidylinositols. Crucially, these genetic determinants not only influence the composition of individual lipids but also provide mechanistic insights into the risk of cardiometabolic disease.

One example is genetic variants in the lipoprotein lipase gene (LPL) that reduce TAG levels, which are associated with a lower risk of CVD and type 2 diabetes (T2D). Similarly, variants in the SPTLC3 gene that reduce Cers are linked to the FADS2 gene, which is crucial for synthesizing FAs associated with peripheral arterial disease.

These genetic determinants not only influence individual lipid composition but also provide mechanistic insights into cardiometabolic disease risk and reveal molecular pathways that can serve as therapeutic targets.

Other key physiological determinants include body mass index (BMI), sex and age [7,8]. BMI is strongly associated with the lipid profile, as increased adiposity is related to alterations in hundreds of lipid species. A specifically higher BMI is associated with an increase in sphingolipids such as Cers and dihydroceramides, as well as a reduction in certain glycosphingolipids and phospholipids, including ether-linked species. These alterations reflect dysregulated lipid homeostasis, commonly associated with a proinflammatory metabolic state.

Sex-related differences are well documented: women tend to exhibit higher plasma concentrations of FAs, SMs, and PCs compared to men [17,18]. In contrast, men generally tend to exhibit higher plasma concentrations of lysophospholipids and TAGs than women. Sex differences in Cer concentrations appear to be influenced by the specific FA side chain composition. These sex-related differences in the human lipidome are driven by a complex interplay of hormonal, genetic, physiological, and life course factors, with significant implications for cardiometabolic risk and disease presentation. Sex hormones (estrogens, androgens, and progestogens) modulate lipid metabolism, influencing the synthesis, distribution, and clearance of lipid species. For example, estrogens are associated with higher HDLc and certain phospholipid species in women, while androgens tend to lower HDLc and increase VLDL TAGs in men. However, the effects of endogenous hormones are complex and not solely responsible for observed differences; exogenous hormone administration does not fully recapitulate these patterns [19,20,21,22]. In addition, life course transitions such as puberty, pregnancy, and menopause in women lead to dynamic changes in lipid profiles. Menopause, for instance, is associated with a shift toward a more atherogenic lipidome, including increased LDLc and TAGs. Additionally, in post-menopause women, disturbances in lipid metabolism, especially in CEs, have also been observed [23].

### 3.2. Metabolic and Inflammatory Conditions

Metabolic and inflammatory conditions profoundly impact the lipidome by inducing both quantitative and qualitative changes in circulating and tissue lipid species. Chronic inflammation, as seen in obesity, metabolic syndrome (MetS), and related disorders, leads to dysregulation of lipid metabolism characterized by increased TAG-rich lipoproteins, reduced HDLc, and accumulation of bioactive lipids such as Cers LPCs. These changes are driven by inflammatory cytokines (e.g., IL-6, TNF-α) and signaling pathways (e.g., NF-κB, inflammasome activation), which alter lipid synthesis, uptake, and remodeling in various tissues [24,25].

Inflammatory processes also promote lipid remodeling within immune cells, affecting membrane composition and generating lipid mediators (e.g., eicosanoids, resolvins) that modulate immune responses and perpetuate inflammation. Dysregulated cholesterol and FA metabolism in immune cells can further amplify inflammatory signaling and contribute to chronic disease progression [25,26,27].

Metabolic stress, such as nutrient excess, leads to lipotoxicity and ectopic lipid accumulation, which disrupts cellular homeostasis and triggers inflammatory cascades. The interplay between lipid metabolism and inflammation is bidirectional: inflammation alters lipid profiles, and specific lipid species can act as upstream regulators of inflammatory pathways, including inflammasome activation and cytokine production [25,28,29].

Dietary patterns and bioactive lipids (e.g., unsaturated fatty acids: UFAs) can modulate the lipidome and inflammatory status, offering therapeutic potential for metabolic and inflammatory diseases. Overall, the lipidome serves as both a target and mediator of metabolic and inflammatory dysregulation, with specific lipid species emerging as biomarkers and therapeutic targets in these conditions [25,28,29].

### 3.3. Gut Microbiota

Emerging evidence highlights the influence of gut microbiota on host lipid metabolism [30]. Specifically, the gut microbiota affects the human lipidome by regulating lipid digestion, absorption, and metabolism through microbial metabolites, bile acid transformation, and modulation of host signaling pathways, with significant implications for metabolic health and disease risk. During colonic digestion, the interplay between host and microbiota results in the production of a diverse array of enzymes, hormones, vitamins, and bioactive compounds, including short-chain fatty acids (SCFAs), bile acids, and conjugated linoleic acids. SCFAs, secondary bile acids, and trimethylamine influence host lipid metabolism by acting on hepatic lipid synthesis, bile acid signaling, and cholesterol transport pathways. For example, gut bacteria can transform primary bile acids into secondary bile acids, which regulate lipid absorption and hepatic cholesterol metabolism via the farnesoid X receptor (FXR) and fibroblast growth factor 19 (FGF19) signaling axes [31,32].

In addition, dietary lipids themselves shape the gut microbiota, which in turn influences host lipid metabolism, creating a bidirectional relationship [33,34]. Lipidomics and metabolomics studies confirm that gut microbiota contributes to interindividual variation in blood lipid levels and can be a target for therapeutic intervention in dyslipidemia and cardiometabolic disease [35].

### 3.4. Lifestyle Factors

Lifestyle behaviors significantly affect lipidomic profiles. Diet quality, physical activity, alcohol consumption, smoking, and adiposity are the principal lifestyle factors that affect the human lipidome.

Both chronic and acute physical activity have been shown to modulate lipid species related to oxidative capacity, energy metabolism and inflammation [36]. Physical inactivity and excess body weight are associated with unfavorable changes in lipid classes, including higher TAGs, lower HDL, and altered phospholipid metabolism, with strong correlations to BMI and insulin resistance [18,37].

Sleep deprivation is another modulator, with studies indicating reduction in plasmalogen choline levels, potentially linked to oxidative stress [38]. Chronic psychological stress and smoking have also been associated with unfavorable lipid changes, including increased proatherogenic lipid accumulation [36,38,39]. In addition, alcohol intake modifies specific lipid species, such as LPCs, and is associated with increased TAGs and altered phospholipid profiles [37,40].

In summary, adherence to healthy lifestyle patterns—characterized by regular physical activity, healthy diet, and avoidance of smoking and excessive alcohol—results in improved lipidomic signatures, including higher HDL function, lower VLDL and TAGs, and reduced inflammatory markers [41,42,43]. In fact, The American Heart Association highlights that early-life nutrition and modifiable lifestyle factors have a major impact on lifelong lipid metabolism and cardiometabolic risk, underscoring the importance of targeting these factors for optimal lipidomic health [44].

### 3.5. Diet and Food Matrix Effects

Diet is one of the most modifiable factors influencing the lipidome [7,45,46]. Not only do weight loss and physical activity shape lipid profiles [47], but molecular features of foods, including the fatty acid composition, supramolecular structure, and the food matrix, also play a pivotal role. These structural characteristics affect fat digestion, absorption, and postprandial lipid metabolism, influencing the release and integration of lipids from other macronutrients such as proteins, sugars, starches, and fibers [48].

## 4. Effect of Diet on Lipidome

Building on this understanding of endogenous and exogenous modulators, the following section will focus on how specific dietary patterns and nutrients influence the plasma, serum and lipoprotein lipidome, with emphasis on their implications for cardiometabolic health.

### 4.1. Plasma Lipidome

Nutritional lipidomics has garnered increasing attention in recent years, with many studies prioritizing plasma as the primary analytical matrix over alternative biological samples, such as serum or isolated lipoprotein fractions. This preference is largely due to plasma’s ability to capture comprehensive diet-induced lipidomic signatures that may serve as early indicators of metabolic health status and cardiometabolic disease risk. Plasma lipid profiles offer a dynamic reflection of both acute dietary intake and chronic dietary patterns, making them particularly valuable for exploring the metabolic effects of nutritional interventions.

#### 4.1.1. Dietary Patterns

Numerous studies have evaluated the impact of different dietary patterns on the plasma lipidome. The PREDIMED (Prevention with Mediterranean Diet) study, a landmark Spanish randomized clinical trial, evaluated the effects of the Mediterranean diet (MedDiet) on the primary prevention of CVD [12,49]. While no significant changes in the overall plasma lipidome were observed between either MedDiet group (MedDiet + extra virgin olive oil -EVOO- or MedDiet + Nuts) and the control group [12], a trend of reductions in lipid species associated with a higher CVD risk, including TAGs, Monoacylglycerols (MAGs), DAGs and PEs, was noted in participants assigned to the MedDiet and EVOO arm. In a follow-up lipidomic analysis [49], participants assigned to the MedDiet + EVOO or MedDiet + nuts groups experienced statistically significant reductions in specific lipid species after one year, compared to the control group. However, after correction for multiple comparisons, only the reduction in cholesterol ester CE (20:3) in the MedDiet + nuts group remained statistically significant.

Beyond the MedDiets, other dietary models have been evaluated. A study investigating the effects of a healthy Nordic diet in individuals with MetS reported increases in plasmalogens and reductions in CE at both 12 and 24 weeks, but not at 18 weeks, suggesting time-dependent lipidomic responses [50]. Emerging evidence also supports the role of low-glycemic load diets in improving cardiometabolic health via lipidomic responses. In the study carried out by Dibay Moghadam et al. [51] participants following a low-glycemic load diet for 28 days had 67 lipid species, predominantly TAG class, elevated compared to those on a high-glycemic load diet, along with three species containing FA 17:0, a potential marker of dairy fat intake. Evidence from other work showed that a healthy diet rich in whole grain, fatty fish and bilberries produced changes in nearly 7% of identified metabolites, including multiple TAGs incorporating long chain (n-3) PUFAs [52].

Intermittent fasting, particularly during Ramadan, has also been studied for its lipidomic effect. In a prospective cohort of 57 overweight and obese adults aged 18–58 years, intermittent fasting was associated with improvements in plasma sphingosine, sphinganine sphingomyelin, and dihydrosphingomyelin species [53].

In subjects with ischemic heart disease, a lacto-ovo-vegetarian diet resulted in lower plasmatic concentration of lipotoxic species, including PC, alkyl-PCs (O-PCs) and saturated TAGs, compared to a meat-based diet, suggesting a reduction in metabolic disease risk [54]. Interestingly, the vegetarian diet also led to increased concentrations of TAGs containing long-chain PUFAs, which were inversely associated with coronary artery disease (CAD) burden.

#### 4.1.2. Dietary Fats

Several studies have investigated the effects of dietary fat quality on plasma lipidomic profiles [55,56,57,58,59]. Recent analysis integrating data from Randomized Controlled Trials (RCTs) and observational cohorts (European Prospective Investigation into Cancer and Nutrition, EPIC-Potsdam cohort, Nurses’ Health Study and the PREDIMED study) suggest that replacing dietary SFAs with plant-based unsaturated FAs yields cardiometabolic benefits, as reflected in improved lipidomic signatures [57]. These studies also indicate that lipidomic-based scores may serve as sensitive markers for metabolic adaptations in response to dietary fat changes. Another study replacing dietary saturated fatty acids (SFAs) with monounsaturated fatty acids (MUFAs) and/or PUFAs demonstrated that high-UFA interventions reduced plasma within-class FAs associated with a higher CVD risk, especially SFA-containing glycerolipids and sphingolipids (e.g., DAG 20:0), whereas they increased those inversely associated with CVD risk [58].

In healthy individuals who consumed foods supplemented with different vegetable oils rich in PUFA, such as sunflower, linseed, echium, and microalgae oil, it was evidenced that linoleic acid and α-linolenic had minimal impact on the species profiles of Glycerophospholipid (GPL), Sphingolipid (SPL), and CE. In contrast, stearidonic acid and docosahexaenoic acid (DHA) were more selectively incorporated into ethanolamine-containing GPL [59]. Alterations of plasma lipidome have also been documented following fish oil supplementation. Compared to a control group receiving high oleic sunflower oil, healthy participants consuming 1.6 g/day of combined eicosapentaenoic acid (EPA) and DHA for three weeks exhibited a more pronounced response in several lipid species, namely PC, PE, LPC, SM, PS, phosphatidylglycerols, and TAGs [56]. In particular, the fish oil group showed increased concentrations of phospholipids and TAGs enriched in long-chain PUFAs. A similar trend was observed in a study by Shabrina et al. [55] in which a 12-week calorie-restricted diet combined with fish oil supplementation led to a two-fold increase in lipid species containing long-chain PUFAs, such as TAG (60:9) and PC (PC 40:6).

In another study, fatty fish intake was shown to reduce lipids that are potential mediators of lipid-induced insulin resistance and inflammation, such as Cer, LPCs and DAGs). Conversely, lean fish consumption was associated with increased concentration of CE and specific long-chain TAGs [60]. Additional evidence suggests that food-derived lipid classes exert a sustained impact on lipid molecular species in healthy women. After four weeks of daily krill oil supplementation, concentrations of some lipid species, including PC, PE, SM, CER and TAG, were increased, especially those containing omega-3-PUFA [61]. To date, two studies have examined postprandial lipidomic responses to dietary fats, one in men [62] and other in women [63]. The study in men demonstrated that plasma phospholipid concentrations varied postprandially depending on the dietary lipid source, such as dairy versus soy-based meals) [62]. The study in women found that EPA and DHA from krill oil were preferentially incorporated into phospholipid molecular species, while EPA and DHA from fish oil were preferentially directed toward neutral lipid species [63]. Another intervention study showed that MUFA-rich and mixed UFA-rich diets reduced lipid species associated with high cardiovascular risk, while increasing those linked to lower risk [64]. Finally, in a randomized controlled feeding study among Chinese adults with overweight and prediabetes, the MedDiet induced distinct plasma lipidomic signatures compared to traditional Chinese and transitional diets, and these changes were associated with modified T2D risk factors [65]. Specifically, 26 lipid species were altered across diet groups, including 12 (TAG fractions, nine plasmalogens, four PCs, and one PE. MedDiet intake was associated with increased TAG fractions and PCs linked to habitual fish consumption, while traditional Chinese diet intake was associated with increased plasmalogens linked to red meat consumption. In terms of diabetes risk, 10 TAG fractions and PC (16:0/22:6) were associated with improved insulin sensitivity (Matsuda index), whereas two plasmalogens were associated with worsened fasting glucose. These findings suggest that fish-related lipidomic changes (TAGs, PCs) reflect beneficial metabolic effects, while red meat-related plasmalogen changes may be detrimental for glycemic control.

#### 4.1.3. Other Food Products

A lipidome-wide outcome analysis of a randomized crossover trial involving individuals with type 1 diabetes (*n* = 10) following a low carbohydrate diet (<100 g carbohydrates/day) revealed increased concentrations of SM (d36:1) and PCs (P-36:4/Phosphatidylcholine-oxide, PCO-36:5), which are believed to exert protective effects against dyslipidemia [66].

Alternative protein sources such as mycoprotein, a low-energy protein- and fiber- rich food derived from fungi, have also demonstrated lipid-modifying effects. A one-week substitution of meat/fish with mycoprotein in healthy adults significatively altered the plasma lipidome [67]. In another study, 16 weeks of adherence to a healthy dietary pattern, with or without wolfberry supplementation, resulted in distinct changes in plasma lipids [68]. Notably, HDLc increased in the wolfberry group and was positively correlated with 10 PC-related lipid species, suggesting a potential mechanism linking this fruit with improved lipid metabolism. Finally, the effects of coffee consumption, a rich source of bioactive compounds such as chlorogenic acids, have also been investigated. After eight weeks of daily coffee intake, participants showed an increase in CEs, and a decrease in oxysterols and free FAs which may suggest improved lipid transport and storage, along with a reduction in oxidative stress and lipid peroxidation [69].

### 4.2. Serum

Like plasma, serum is one of the preferred matrices in lipidomic studies due to its accessibility, standardized collection protocols, and ability to capture a broad spectrum of circulating lipid species relevant to metabolic, cardiovascular, and other systemic diseases. Plasma and serum lipidomics workflows have been extensively optimized to achieve high coverage, reproducibility, and throughput, enabling the quantification of hundreds or thousands of lipid species across multiple subclasses using advanced LC-MS/MS platforms and stable isotope dilution techniques [70,71,72].

In an in-depth serum lipidomics study involving 104 overweight participants with prediabetes, an 8-week low-energy diet resulted in a reduction in DAGs, Cers, LPCs, and ether-linked PEs, along with an increase in ether-linked PCs [73]. Another study evaluated the relationship between Diet Quality Indices, including the Healthy Eating Index (HEI)-2015, the Alternate HEI-2010 (AHEI-2010), and the alternate Mediterranean Diet Index (aMED), and serum lipidomic profiles in two cohorts of middle-aged and older men from Finland and the U.S. (*n* = 1338) [74]. Higher adherence to these indices was associated with favorable shifts in lipidomic profiles, mostly TAGs or FA 22:6-containing species, which are related to seafood and plant protein intake, as well as dietary EPA-DHA,. Additional evidence from participants with and without diabetes showed that HEI adherence was significantly associated with differences in serum lipidome, reinforcing the influence of healthy dietary patterns on metabolic concentrations [75]. In patients with rheumatoid arthritis (*n* = 50), a 10-week intervention comparing a Mediterranean-style diet versus a Western-style control diet revealed that MedDiet improved the serum lipid profile, shifting it toward a less atherogenic pattern [76]. Specifically, favorable changes were observed in TAGs, CEs, PCs, alkylphosphatidylcholines and alkenylphosphatidylcholines.

Beyond dietary patterns, several studies have focused on the impact of specific foods and bioactive compounds. For example, in a study involving 90 women at high cardiovascular risk, the effects of consuming olive oil, camellia seed oil, and soybean oil were compared. The most significant differences in serum lipid composition were found in LPC and PE classes [77]. In another study, 13 weeks of n-3 FA supplementation (6 g/d) in patients with Human Immunodeficiency Virus (HIV), elevated triglycerides, and/or insulin resistance resulted in a reduction in arachidonic acid in the phospholipid fraction [78]. Another 12-week, randomized, double-blind, placebo-controlled trial involving 309 Chinese patients with T2D and hypertriglyceridemia found that fish oil (mix of EPA and DHA) intake led to significant elevations in DHA and EPA, along with lipids containing n-3 PUFA acyl chains [79]. Notably, significant reductions were observed in TAGs, PCs, PEs, Cers, and LPCs and most of these reduced lipids contained low-unsaturated or saturated acyl chains, such as 18:1, 18:2, 18:0, and 16:0. Additionally, a reduction in the levels of arachidonic acid, a proinflammatory metabolite, was observed.

Until now, it is well known that plant sterols reduce total cholesterol and LDLc. One study assessed the effects of the consumption of plant-sterol enriched yogurt drinks with different fat contents (0.1% vs. 1.5% milk fat) [80]. The yogurt with 0.1% milk fat led to greater lipidomic changes, including reductions in several SMs, which correlated well with LDLc reduction, and decreases in LPCs (LPC (16:1), LPC (20:1)), and cholesterol arachidonate, all associated with reduced inflammation and atherogenic potential. In a quantitative targeted metabolomic study involving 284 male participants, coffee consumption was positively associated with two classes of SMs and negatively associated with medium- and long-chain acylcarnitines, indicating possible lipid metabolism modulation through coffee-derived bioactive [81]. Finally, in a study involving athletes, responders to a DAG diet intervention, defined by normalization of serum uric acid, showed notable changes in plasmalogen PCs and TAGs compared to non-responders [82], suggesting a lipidomic shift associated with metabolic responsiveness.

### 4.3. HDL Lipidome

HDL plays a crucial role in lipid metabolism and cardiovascular protection, extending beyond their classical function in reverse cholesterol transport. The HDL lipidome is highly complex and dynamic, influenced by factors such as diet, age, gender, metabolic conditions, and some pharmacological treatments like statins, all of which can affect HDL functionality, cardiovascular risk, and immune responses [7,45,46,47,83]. This specific lipid composition of HDL particles is tightly linked to their functional properties, including antioxidant, anti-inflammatory, and endothelial-modulating activities. Accordingly, studying the HDL lipidome offers valuable insights into HDL functional quality and its relevance to metabolic diseases such as T2D and atherosclerosis.

In a recent longitudinal crossover clinical study involving overweight subjects, it was shown that the HDL lipid pattern is diet dependent. Notably, dairy-based omega-3 supplementation led to more pronounced changes, especially in CEs, than phytosterols [7]. Similarly, a study in hypertriglyceridemic patients showed that five weeks of enriched HDL particles with EPA and DHA supplementation significantly increased these FAs within HDL at expenses of n-6 FA such as arachidonic acid [45]. Consistent effects were observed in healthy young adults (*n* = 7) in whom 30 days of fish oil supplementation (1125 mg EPA and 875 mg DHA per day) led to a significant increase in 31 HDL-associated phospholipids species [84].

The phenolic compounds of virgin olive oil have also been shown to affect HDL lipid profiles. In a randomized, controlled, double-blind, crossover trial involving individuals with hypercholesterolemia, supplementation with olive oil for only three weeks significantly modified TAG species in HDL, suggesting their responsiveness to phenolic content [46]. The effects of whole egg versus yolk-free egg consumption on HDL lipid pattern were evaluated in overweight, postmenopausal women [85] revealing a significant increase in the proportion of PE-HDL with whole egg consumption compared with yolk-free egg.

Another study comparing the effects of different dietary patterns demonstrated that lipid classes, such as PC, TAG and CE, were more sensitive to dietary influences, whereas other lipid classes (Cers and SM) reflected non-dietary factors [86]. Notably, a fast-food diet increased HDL content of PE, SFA and odd chain FA, whereas the MedDiet led to an enrichment of very-long chain FA and UFA in HDL. Beyond dietary components, a study by Khan et al. [47] explored the impact of weight loss and exercise on HDL lipidome. At baseline, individuals with MetS displayed significant lower levels of sphingolipids and phospholipids, and higher levels of lysoalkylphosphatidylcholine, lysophosphatidylinositol, phosphatidylglycerol, DAGs and TAGs, compared to healthy controls. Following intervention, the HDL lipidome normalized, particularly in dihexosylceramide and phosphatidylinositol species, suggesting that specific lipid classes may serve as biomarkers or mechanistic contributors to metabolic dysfunction.

### 4.4. LDL Lipidome

LDL plays central roles in cholesterol transport and metabolic homeostasis yet paradoxically represents the primary lipid fraction driving atherogenesis. This dual physiological-pathological nature arises from complex interactions between its lipid composition (CEs, phospholipids, and TAGs), and the structure of apolipoprotein B-100, which together influences its potential for vascular deposition [87]. Beyond CVD, LDL’s biological significance extends to immunomodulation, inflammation, and even neurodegenerative processes, particularly through pathways involving oxidized lipids [88,89].

In a study by Egert et al. [90], the effects of six-week consumption of margarines enriched with α-linolenic acid (ALA), EPA, or DHA on the LDL lipidome were examined in a group of normolipidemic, non-obese volunteers. The intervention significantly enriched LDL particles with the respective FAs, revealing that isolated dietary n-3 FAs intake leads to differential incorporation into LDL, partially due to interconversion. Specifically, ALA supplementation increased EPA levels by 36%; EPA supplementation led to a further 24% increase in DHA; and DHA supplementation surprisingly resulted in a 249% increase in EPA.

Lipidomic changes in LDL were also assessed in subjects with impaired glucose metabolism after consumption of *Camelina sativa* oil, fatty fish and lean fish [91]. In this randomized controlled trial, *Camelina sativa* oil led to a decrease in saturated and monounsaturated CE species and an increase in ALA-containing ω3-long chain-PUFAs. In overweight, moderately hypercholesterolemic individuals, phytosterol intake was associated with a reduction in LDL-glycerophospholipids, with notable changes in PC and PS subclasses. Additionally, ω3–enriched milk intake over four weeks significantly increased long-chain polyunsaturated CEs [92].

Despite the recognized biological importance of LDL, only three studies have systematically investigated how dietary factors affect its lipid composition. While these studies have consistently demonstrated changes in PUFA profiles in response to dietary interventions, evidence regarding other dietary sources and lipid classes remains limited.

## 5. The Relevance of Lipidome in Health and Disease

Recent human studies have shown that the plasma lipidome undergoes marked alterations during transitions from health-to-disease [11,12,13,93,94,95]. These shifts are particularly pronounced in metabolic disorders such as obesity, T2D, NAFLD, and atherosclerosis. As bioactive molecules involved in energy storage, membrane architecture, and cellular signaling, lipids play a critical role in maintaining metabolic balance. Dysregulation in lipid synthesis, storage, and signaling disrupt cellular homeostasis and systemic metabolism, contributing to the pathogenesis of these conditions. The most relevant studies examining the associations between lipidomic profiles and CVD risk and mortality are outlined below.

The Bruneck Study incorporated comprehensive lipidomic profiling over a 10-year follow-up period (2000–2010) to investigate associations between circulating lipid species and CVD risk [11]. Multiple lipid classes, including CEs, LPCs, PCs, PEs, SMs, and TAGs, were significantly linked to CVD incidence. Notably, the lipid species with the strongest predictive value were TAG (54:2), CE (16:1), and PE (36:5), characterized by low carbon number and double-bond content, highlighting specific lipidomic signatures associated with CVD pathogenesis.

The Cardiovascular Health Study [93] investigated the associations between Cer and SM species, with varying saturated fatty acid chain lengths, and heart failure risk in 4249 participants. Elevated plasma concentrations of Cer-16 and SM-16 were associated with an increased risk of heart failure, while higher concentrations of Cer-22, SM-20, SM-22, and SM-24 were linked to reduced risk, indicating a chain-length-dependent association with cardiovascular outcomes.

In the Ludwigshafen Risk and Cardiovascular Health (LURIC) study [94], lipidomic analyses revealed that highly polyunsaturated PC species, LPC species, and long chain saturated SM and Cer species were associated with a protective effect. In contrast, saturated and monounsaturated PC species, particularly PC 32:0 and palmitate containing SM and Cer species, were strongly positive associated with all-cause mortality.

The PREDIMED trial provided further insight into lipidomic predictors of CVD. In a sub-cohort of 787 participants, including 230 incident CVD cases [95] hydroxylated phosphatidylcholine (HPC) cluster lipids and other cluster including DAGs and a MAG with a stearic acyl chain were the most strongly associated with increased cardiovascular risk were. A one-standard deviation increments in the HPC cluster score corresponded to a hazard ratio of 1.39 (95% Confidence Interval, CI, 1.17–1.66) for CVD, while the DAG/MAG cluster had a hazard ratio of 1.24 (95% CI, 1.11–1.37). Conversely, clusters rich in highly unsaturated phospholipids and CEs were inversely associated with cardiovascular risk. These findings highlight that lipid clusters with fewer double bonds (i.e., more saturated or less unsaturated) and specific structural features, such as hydroxylation or the presence of stearic acid, are linked to higher cardiovascular risk, while greater unsaturation in lipid species is associated with lower risk. An additional study, within PREDIMED trial, demonstrated that MAGs/DAGs and short TAGs showed a direct association, while PC/LPC/PC-plasmalogens and polyunsaturated CEs were inversely associated with CVD [12]. Other analyses within the same PREDIMED cohort confirmed a positive relationship between baseline plasma Cer concentrations and incident CVD [13].

In the BioHEART-Computed Tomography (CT) Discovery Cohort [93], lipidomic profiling of plasma from 994 participants showed that acylcarnitine, PC as well as Cer species played pivotal roles in the development of CADs.

The mechanistic link between these lipids and cardiovascular risk can therefore be summarized as follows: Cers aggravate cardiovascular risk by promoting endothelial dysfunction, oxidative stress, vascular inflammation, and atherogenesis through both direct cellular effects and receptor-mediated signaling pathways [96,97,98]. On the other hand, the mechanism by which TAGs contribute to cardiovascular risk is through the atherogenicity of triglyceride-rich lipoproteins (TRLs) and their remnants, which promote intimal cholesterol deposition, inflammation, and endothelial dysfunction, ultimately driving atherosclerosis and cardiovascular events [99,100,101].

Regarding PCs, mechanistically, PCs influence cardiovascular risk through several pathways. First, dietary PC is metabolized by gut microbiota to produce trimethylamine (TMA), which is then oxidized in the liver to trimethylamine-N-oxide (TMAO). Elevated plasma TMAO is associated with increased risk of major adverse cardiovascular events, as demonstrated in large prospective cohorts. TMAO promotes atherogenesis by upregulating macrophage scavenger receptors, enhancing foam cell formation, and impairing cholesterol metabolism [102,103]. Second, the plasma PC profile itself is independently associated with cardiovascular outcomes. Lower baseline levels of total choline-containing phospholipids predict higher risk of all-cause and cardiac mortality in patients with acute coronary syndromes. Conversely, higher levels of polyunsaturated PC species are inversely associated with incident CVD and mortality, suggesting a protective effect [94].

Overall, the plasma SM profile and its species composition are emerging as important metabolic signatures and mechanistic contributors to cardiovascular risk, with both pro-atherogenic and potentially protective roles depending on molecular structure. In this sense, the plasma profile of SMs is characterized by their presence predominantly in atherogenic lipoproteins (especially LDL and VLDL) [104,105].

## 6. Conclusions and Future Directions

A growing body of evidence demonstrates that dietary interventions can significantly modify the human lipidome, highlighting the potential of lipidomics as a tool for understanding metabolic response to diet. However, no single lipid species has emerged as a consistent marker or mediator of these effects across diverse populations or dietary patterns. Instead, observed changes tend to affect multiple lipid classes simultaneously, indicating a systemic modulation of lipid metabolism rather than isolated effects on individual lipid molecules.

Despite promising findings, there remains no clear consensus regarding the optimal dose, duration, or type of intervention required to induce favorable and sustained lipidomic shifts. This lack of standardization limits the translation of lipidomic data into specific, evidence-based dietary recommendations. Therefore, future research should prioritize the design and execution of systematic, well-controlled and standardized studies aimed at identifying the most effective dietary strategies for modifying the lipidome. Moreover, greater focus should be placed on elucidating the biochemical and functional mechanisms linking diet-induced lipidomic alterations to clinical meaningful outcomes. Such efforts will be crucial for integrating lipidomics into the framework of precision nutrition, ultimately supporting personalized prevention and treatment strategies in cardiometabolic health.

## Figures and Tables

**Table 1:** Classification of lipids and representative examples.

Lipid Category	Examples
Fatty acyls	FAs (e.g., palmitic acid, arachidonic acid), eicosanoids.
Glycerolipids	Mono-, di-, and triacylglycerols
Glycerophospholipids	PCs, PEs, LPCs
Sphingolipids	Cers, SMs and glycosphingolipids.
Sterol lipids	CEs
Prenol lipids	Isoprenoids such as ubiquinone (coenzyme Q), dolichol.
Saccharolipids	Lipid A (component of bacterial lipopolysaccharide).
Polyketides	Erythromycin, tetracycline (antibiotic polyketides).

FA: Fatty acids; PCs: Phosphatidylcholines; Pes: phosphatidylethanolamines; LPCs: lysophosphati-dylcholines; Cers: Ceramides; SMs: Sphingomyelins; CEs: Cholesterol esters.

## Data Availability

Not applicable.
